# The role of long noncoding RNAs in therapeutic resistance in cervical cancer

**DOI:** 10.3389/fcell.2022.1060909

**Published:** 2022-11-09

**Authors:** Meimei Zhou, Linlin Liu, Jing Wang, Wanru Liu

**Affiliations:** Center for Reproductive Medicine, Center for Prenatal Genetics, First Hospital of Jilin University, Changchun, Jilin, China

**Keywords:** lncRNAs, cervical cancer, resistance, mRNA, miRNAs, therapy

## Abstract

Cervical cancer is one of the common tumors and often causes cancer-related death in women. Chemotherapy is a common cancer therapy, which displays a pivotal clinical benefit for cancer patients. However, chemoresistance becomes a big obstacle for failure of treatment in cancer patients. Recently, long noncoding RNAs (lncRNAs) have been identified to regulate drug resistance in human cancers, including cervical cancer. In this review, we describe the role of lncRNAs in regulation of chemotherapeutic resistance in cervical cancer. We also discuss the molecular mechanisms of lncRNA-mediated drug resistance in cervical cancer. Moreover, we describe that targeting lncRNAs could reverse drug resistance in cervical cancer. Therefore, lncRNAs could become effective therapeutic targets and chemotherapeutic sensitizers for cervical cancer patients.

## Introduction

Cervical cancer is one of the common tumors and often causes cancer-related death in women (Sung et al., 2021; Siegel et al., 2022). Human papillomavirus (HPV) infection is the common cause for cervical oncogenesis (Okunade et al., 2022). The treatment for cervical cancer includes surgery, radiation, chemotherapy or combination therapies. Chemotherapy is a common cancer therapy for killing tumor cells (Miller et al., 2022). Chemotherapy has shown a pivotal clinical benefit for cancer patients. However, drug resistance becomes a key reason for failure of treatment in cancer patients. Many factors are associated with drug resistance in human cancers, including oncogenic action, inactivation of tumor suppressor genes, impaired DNA repair, cancer stem cells (CSCs), epithelial to mesenchymal transition (EMT) and exosomes (Friedmann Angeli et al., 2019; Marine et al., 2020; Bayik and Lathia, 2021; Passaro et al., 2021; Weiss et al., 2022). Multidrug resistance protein (MRP)-related ABC family includes ABCC11, ABCC12, MRP1-7 proteins. The G family member, ABCG2/BCRP, is also associated with drug resistance (Haimeur et al., 2004). EMT is a biological process: epithelial cells become mesenchymal cells after specific stimulations *via* loss of their cell polarity and adhesion and acquirement of invasive and migratory ability (Lambert and Weinberg, 2021). CSCs are specific cells with abilities of self-renewal, tumorigenicity and differentiation, which often have biomarkers of CSCs (Walcher et al., 2020). The tumor microenvironment (TME), which includes blood vessels, extracellular matrix, stromal cells and immune cells, is also involved in chemoresistance in cancer patients (Liu et al., 2022a; Barnestein et al., 2022; Balaji et al., 2021). Recently, noncoding RNAs (ncRNAs) have been identified to take part in drug resistance and immune resistance in human cancer (Jiang et al., 2020; Jiang et al., 2021; Xie et al., 2022; Zhou et al., 2022).

Long non-coding RNAs (lncRNAs) belong to non-coding RNA family and have larger than 200 nucleotides (Liu et al., 2021). Noncoding RNAs often have no functions for translation into proteins; however, noncoding RNAs play a pivotal role in epigenetic regulation (Statello et al., 2021; Zheng et al., 2022). Numerous studies suggested that lncRNAs are critically involved in carcinogenesis and tumor development in a variety of human malignancies (Liu et al., 2020a; Chen et al., 2022a; Liu and Shang, 2022). LncRNAs have been identified to participated in cervical carcinogenesis and progression *via* regulation of proliferation, autophagy, differentiation, apoptosis, invasion, migration, and metastasis (Cui et al., 2017; Aalijahan and Ghorbian, 2019; Caceres-Duran et al., 2020; Tornesello et al., 2020). LncRNAs could be prognostic and diagnostic biomarkers in cervical cancer patients (Chi et al., 2017). Moreover, circulating lncRNAs might be biomarkers for prediction of cervical cancer prognosis (Sun et al., 2018). In this review, we describe the role of lncRNAs in regulation of chemotherapeutic resistance and radio-resistance in cervical cancer. We also discuss the molecular mechanisms of lncRNA-mediated drug resistance in cervical cancer. Moreover, we describe that targeting lncRNAs could reverse drug resistance in cervical cancer. Therefore, lncRNAs could become effective therapeutic targets and chemotherapeutic sensitizers for cervical cancer patients.

## LncRNAs regulate therapeutic resistance

Mounting evidence has demonstrated that numerous lncRNAs control the therapeutic resistance in human cancers, including chemoresistance, radio-resistance and immune-resistance. In the following sections, we describe how lncRNAs govern therapeutic resistance in cervical cancer.

## LncRNA HOTAIR in cervical cancer

### HOTAIR expression and clinical features of cervical cancer patients

Evidence revealed that lncRNA HOTAIR plays an essential role in tumorigenesis and progression in human cancers. Studies have showed that upregulation of HOTAIR was linked to tumor malignant progression (Hajjari and Salavaty, 2015; Kim et al., 2015; Sun et al., 2016; Zhou et al., 2020). One study measured the lncRNA HOTAIR expression levels in 218 cervical cancer samples and the matched adjacent normal samples by quantitative RT-PCR (Huang et al., 2014). This study found that HOTAIR expression was increased in cervical cancer tissues and correlated with age, FIGO stage, invasion and lymph node and tumor size (Huang et al., 2014). Moreover, high expression of HOTAIR was associated with worse OS and DFS in cervical cancer patients. Furthermore, HOTAIR expression level can act as an independent biomarker for OS in cervical cancer patients (Huang et al., 2014). Another study measured the circulating HOTAIR by QRT-PCR in the sera of 118 cervical cancer patients and 100 normal age-matched females (Li et al., 2015). The expression of HOTAIR in the sera of cancer patients was increased, which was correlated with invasion, lymphatic node metastasis, advanced tumor stages, tumor recurrence and short OS (Li et al., 2015).

### HOTAIR regulates proliferation, migration and invasion

*In vitro* experiments showed that depletion of HOTAIR blocked proliferation, invasion and migration of cervical cancer cells (Kim et al., 2015). HOTAIR promoted cell motility and metastasis *via* modulation of MMP-9, VEGF and EMT-related genes in cervical cancer (Kim et al., 2015). HOTAIR overexpression increased cell growth and invasion *via* modulation of Notch signaling pathway in cervical cancer (Lee et al., 2016). Upregulation of HOTAIR regulated Notch-Wnt signaling pathway and EMT and caused induction of tumor growth *in vitro* and *in vivo* in cervical cancer (Lee et al., 2016). Another study revealed that HOTAIR upregulation stimulated cell viability, invasion, migration, and reduced apoptosis *via* modulation of HLA-G expression by sponging miR-148a in cervical cancer (Sun et al., 2016). Ji et al. (2018) reported that HOTAIR depletion reduced invasion, migration and proliferation *via* sponging miR-17-5p in cervical cancer. Liu et al. (2018) found that lncRNA HOTAIR facilitated tumor progression *via* regulation of BCL-2 expression by sponging miR-143-3p in cervical cancer cells. Zheng et al. (2018) observed that lncRNA HOTAIR elevated invasion and migration of cervical cancer cells *via* inhibition of miR-206 and modulation of megakaryoblastic leukemia 1 (MKL1) in HeLa cells. Moreover, silencing of HOTAIR retarded proliferation, invasion and migration of cervical cancer cells, and STAT3 promoted the activity of the HOTAIR. This work indicated that blockade of HOTAIR and STAT3 synergistically repressed invasion, migration and viability of cervical cancer cells (Zhang et al., 2018a). Again, lncRNA HOTAIR accelerated cell invasion, proliferation and migration *via* modulation of MAPK1 expression in DoTc2 cervical cancer cells (Liu et al., 2020b). Depletion of both HOTAIR and MAPK1 led to suppression of cell growth synergistically in DoTc2 cells (Liu et al., 2020b). Downregulation of HOTAIR inactivated Wnt/β-catenin signaling pathway and reduced the expression of several mRNAs, such as MAGI2, AJAP1, SOX17, PCDH10 and TET1 in cervical HeLa cells (Salmeron-Barcenas et al., 2019). Zhou et al. (2021) discovered that lncRNA HOTAIR inhibited apoptosis and enhanced proliferation *via* reduction of miR-214-3p expression and inducing Wnt/β-catenin signaling pathway in HPV16 positive cervical cancer cells.

### HOTAIR is associated with HPV

LncRNAs have been known to associate with HPV infection in cervical cancer (Liu et al., 2022b; Chen et al., 2022c). HPV16 E7 oncoprotein can modulate the expression of HOTAIR in cervical cancer cells, suggesting that E7 oncoprotein was linked to lncRNA HORAIR expression in cervical cancer (Sharma et al., 2015). Prior study also revealed that genetic variation in the HOTAIR was correlated with HPV16 cervical cancer (Sharma Saha et al., 2016). This work found that rs2366152C was highly represented in HPV positive cervical cancer patients with lower expression of HOTAIR and higher expression of miR-22 (Sharma Saha et al., 2016). Moreover, miR-22 targeted rs2366152C of lncRNA HOTAIR, resulting in downregulation of lncRNA HOTAIR in cervical cancer cells (Sharma Saha et al., 2016). HPV16 E7 reduced the expression of HOTAIR, p53 and NRP2, but increased miR-33-3p, contributing to suppression of cell apoptosis and induction of cell proliferation in cervical cancer (Zhang et al., 2018b). LncRNA HOTAIR sponged miR-331-3p and elevated the expression of NRP2 in HPV positive cervical cancer, which established a negative feedback loop to govern the apoptosis of cervical cancer cells (Zhang et al., 2018b).

### HOTAIR SNP in cervical cancer

Qiu et al. (2016) reported that HOTAIR single nucleotide polymorphism (SNP) rs920778 (C>T) was correlated with cervical cancer risk. Specifically, rs920778 genotype was analyzed in 215 cervical cancer patients and 430 normal controls and observed that TT genotype of rs920778 was associated with HOTAIR upregulation. TT genotype of rs920778 was correlated with higher risk of cervical cancer, advanced tumor stage, tumor grade, HPV infection and lymph node metastasis (Qiu et al., 2016). Cervical cancer patients with TT genotype exhibited resistance to the treatment of EBRT + ICBT + cisplatin, suggesting that rs920778 was linked to drug resistance in cervical cancer patients (Qiu et al., 2016). Guo et al. (2016) also revealed that SNP rs920778T was associated with cervical cancer predisposition *via* regulation of HOTAIR expression. TT genotype was correlated with TNM stage in cervical cancer patients, suggesting that TT genotype of rs920778 might be an indicator of cervical cancer progression (Guo et al., 2016). Similarly, Weng et al. (2018) uncovered that lncRNA HOTAIR SNP rs920778 was linked to worse OS and poorer cancer recurrence in cervical cancer patients. HOTAIR rs7958904 polymorphism has been found to be associated with increased cervical cancer risk (Jin et al., 2017). The rs7958904 CC genotype was linked to higher expression of HOTAIR and promotion of cell growth in cervical cancer (Jin et al., 2017).

### HOTAIR regulates chemo-and radio-resistance

HOTAIR facilitated aggressive behaviors of cervical cancer and induced radio-resistance through inhibition of p21 (Jing et al., 2015). Upregulation of HOTAIR reduced apoptosis and enhanced proliferation, migration and invasion and promoted cell cycle in cervical cancer cells. Depletion of HOTAIR increased the expression of p21 and promoted the radio-sensitivity of C33A cervical cancer cells (Jing et al., 2015). *In vivo* data also showed that stable silencing of HOTAIR repressed tumor growth and increased the sensitivity of radiotherapy (Jing et al., 2015). Li et al. (2018) found that radiotherapy suppressed tumor growth in mice *via* downregulation of HOTAIR expression and HIF-1α in cervical cancer. Upregulation of HOTAIR abolished the efficacy of radiation on apoptosis and viability in C33A and HeLa cells through upregulation of HIF-1α (Li et al., 2018). Depletion of HIF-1α abrogated HOTAIR-induced viability of HeLa and C33A cells, revealing that radiotherapy suppress cell growth *via* regulation of HOTAIR/HIF-1α pathway in cervical cancer (Li et al., 2018). Cisplatin-resistant cells displayed stem cell features and increased migration and invasion, which had the upregulation of HOTAIR (Zhang et al., 2021). Downregulation of HOTAIR reduced the expression of stemness markers in cervical cancer. Notably, lncRNA HOTAIR facilitated cancer cell stemness *via* interaction with miR-203 and induction of ZEB1 expression in cervical cancer (Zhang et al., 2021). One direct evidence revealed that lncRNA HOTAIR enhanced chemoresistance *via* inducing EMT and modulating miR-29b/PTEN/PI3K pathway in cervical cancer (Zhang et al., 2022). HOTAIR increased the resistance of cisplatin, paclitaxel and docetaxel *via* sponging miR-29b in cervical cancer cells (Zhang et al., 2022).

## LncRNA PVT1

LncRNA PVT1 has been known to play a critical role in oncogenesis and progression in a variety of human cancers (Lu et al., 2017; Pan et al., 2018; Wang et al., 2019; Bohosova et al., 2021). Evidence has dissected that lncRNA PVT1 could be chemotherapy and radiotherapy sensitizer in cancer (Yao et al., 2022). Iden et al. (2016) reported that high expression of lncRNA PVT1 was correlated with poor prognosis and cancer phenotype in cervical cancer. Knockdown of PVT1 reduced proliferation, invasion, migration, and increased cisplatin cytotoxicity. The expression of PVT1 was upregulated in cervical cancer cells after hypoxia and immune response stimulation (Iden et al., 2016). Studies have revealed that lncRNA PVT1 sponged miR-195 and regulated EMT in cervical cancer cells, resulting in chemoresistance (Shen et al., 2017). Depletion of HPV16 E7 reduced the expression of PVT1 and restored the expression of miR-195. LncRNA PVT1 can bind with EZH2 and miR-195, and increase the expression level of H3K27me3. Interestingly, overexpression of miR-195 also repressed the expression of PVT1 in cervical cancer cells (Shen et al., 2017). Importantly, PVT1 blocked paclitaxel-induced EMT and sensitized cervical cancer cells to paclitaxel treatment. This study implied that PVT1 modulated paclitaxel resistance of cervical cancer cells *via* regulating miR-195 and EMT (Shen et al., 2017).

## LncRNA UCA1

LncRNA urothelial cancer associated 1 (UCA1) plays a necessary role in carcinogenesis and malignant development (Wang et al., 2017a; Yao et al., 2019a; Xuan et al., 2019). Wang et al. (2017b) reported that lncRNA UCA1 enhanced cisplatin resistance in cervical cancer. LncRNA UCA1 upregulation stimulated cell proliferation, reduced apoptosis and increased cisplatin resistance. Downregulation of UCA1 decreased cisplatin resistance in cervical cancer cells (Wang et al., 2017b). LncRNA UCA1 reduced the expression of caspase 3 and upregulated the expression of CDK2, leading to suppression of apoptosis of cervical cancer cells. UCA1 upregulated the expression of p21 and inhibited survivin expression level and caused enhancement of cell proliferation in cervical cancer cells (Wang et al., 2017b). Together, lncRNA UCA1 promoted cisplatin resistance in cervical cancer, suggesting that blockade of lncRNA UCA1 is a useful approach for cervical cancer therapy.

## LncRNA GAS5

LncRNA growth arrest-specific transcript 5 (GAS5) acts as a pivotal tumor suppressor in various types of human cancers (Ma et al., 2016; Yang et al., 2020; Filippova et al., 2021; Kaur et al., 2022). LncRNA GAS5 has been verified to work as a tumor suppressor to control cisplatin resistance *via* targeting miR-21 in cervical cancer (Wen et al., 2017). LncRNA GAS5 overexpression repressed proliferation, invasion and migration of cervical cancer cells *in vitro* and *in vivo*. The low expression of GAS5 was detected in cervical cancer patients, which was associated with high expression of miR-21 (Wen et al., 2017). LncRNA GAS5 inhibited the expression of miR-21 in cervical cancer cells. Overexpression of lncRNA GAS5 promoted the sensitivity of cisplatin-resistant SiHa cells to cisplatin therapy. Mechanistically, GAS5 inhibited miR-21 expression and elevated the expression of PTEN and influenced the pAkt in cervical cancer cells, leading to suppression of cisplatin resistance (Wen et al., 2017). Yao et al. (2019b) found that lncRNA GAS5 attenuated cisplatin-mediated cell apoptosis through regulation of STAT3 pathway *via* targeting miR-21 in cervical cancer cells. Overexpression of GAS5 induced G0/G1 phase arrest, reduced colony formation and proliferation, attenuated migratory and invasive ability in cervical cancer cells. Moreover, overexpression of GAS5 reduced TIMP3 and PDCD4 expression and inactivated STAT3 and E2F3 in cervical cancer (Yao et al., 2019b). Similarly, Fang and coworkers found that low expression of lncRNA GAS5 could predict cisplatin resistance and worse survival in cervical cancer patients (Fang et al., 2020). Hence, lncRNA GAS5 might be a potential target for reversing cisplatin resistance in cervical cancer.

## Other LncRNAs regulate drug resistance

The expression of lncRNA CASC2 was decreased in cervical cancer samples, which was related with poor prognosis and a shorter OS (Feng et al., 2017). Exogenous CASC2 amplified the repression of proliferation of cervical cancer cells induced by cisplatin treatment. Cisplatin-resistant cervical cancer patients had lower expression of lncRNA CASC2 (Feng et al., 2017). Upregulation of CASC2 increased the cisplatin sensitivity in cisplatin-resistant cervical cancer cells by suppression of miR-21 and upregulation of PTEN and inactivation of pAkt (Feng et al., 2017). LncRNA LINP1 caused DSBs repair *via* regulation of NHEJ pathway and reduced the sensitivity of cervical cancer cells to ionizing radiation (Wang et al., 2018a). Depletion of LINP1 elevated the expression levels of cleaved caspase 3 and PARP, contributing to cell apoptosis in cervical cancer cells after radiation (Wang et al., 2018a). LncRNA MALAT1 facilitated cisplatin resistance *via* modulating the PI3K/AKT pathway in cervical cancer (Wang et al., 2018b). Silencing of MALAT1 increased the expression of cleaved caspase-3, while upregulation of MALAT1 increased the mRNA level of BRWD1 and elevated the expression of p-PI3K and p-Akt in cervical cancer cells (Wang et al., 2018b). Upregulation of lncRNA ZFAS1 was observed in cervical cancer specimens and correlated with poor prognosis (Feng et al., 2019). ZFAS1 siRNA treatment led to suppression of migration, invasion and growth in cervical cancer cells. ZFAS1 siRNA also enhanced cisplatin sensitivity in cervical cancer cells and in nude mice (Feng et al., 2019).

LINC00511 downregulation blocked cell growth *via* reduction of MRP1, P-GP, MMP-2, MMP-9 and Bcl-2 and induction of cleaved caspase-3 and Bax expressions (Mao et al., 2019). Inhibition of LINC00511 increased cancer cell sensitivity to paclitaxel treatment in cervical cancer (Mao et al., 2019). LncRNA TUG1 expression was upregulated in cisplatin-resistant cervical cancer tissues and linked to a worse prognosis (Wei et al., 2019). TUG1 depletion inhibited the expression of RFX7 in cervical cancer cells and enhanced cisplatin sensitivity by activation of the MAPK pathway (Wei et al., 2019). Exosomal lncRNA HNF1A-AS1 enhanced cisplatin resistance *via* elevating the expression of TUFT1 by sponging microRNA-34b axis in cervical cancer cells (Luo et al., 2019). LncRNA miR503HG increased the cisplatin sensitivity *via* suppression of miR-155 and upregulating caspase-3 in recurrent cervical cancer (Zhao et al., 2020). LncRNA NNT-AS1 expression was upregulated in cisplatin-resistant cervical tumors. Silencing of NNT-AS1 attenuated cisplatin resistance *via* directly binding to miR-186 and subsequently increasing HMGB1 expression in cervical cancer (Liu et al., 2020c). Suppression of lncRNA NEAT1 increased the sensitivity of 5-FU *via* sponging miR-34a and elevating the expression of LDHA in cervical cancer cells (Shao et al., 2021). LDHA is a glycolysis key enzyme and regulates glycolysis rate, suggesting that lncRNA NEAT1 increases 5-FU resistance *via* regulation of glycolysis rate in cervical cancer cells (Shao et al., 2021). LncRNA DLG1-AS1 facilitated gemcitabine resistance *via* targeting miR-16-5p and modulating HDGF expression in cervical cancer cells (Zou et al., 2022). Repression of DLG1-AS1 inhibited proliferation of gemcitabine-resistant cervical tumor cells (Zou et al., 2022). Linc00958 was highly expressed in cisplatin-resistant SiHa cells. Moreover, linc00958 expression was correlated with a short survival in cervical cancer patients. Mechanistically, linc00958 regulated miR-185-5p and RSF-1 and modulated cisplatin resistance in cervical cancer cells *via* targeting AKT1/GSK3β/VEGFA pathway (Tian et al., 2022). Depletion of lncRNA NCK1-AS1 promoted cisplatin sensitivity to cisplatin *via* modulating miR-134-5p and restoring the expression of MSH2 in cervical cancer (Zhang et al., 2019). Altogether, lncRNAs regulate the chemotherapeutic resistance in cervical cancer treatment.

## Conclusion

**FIGURE 1:**
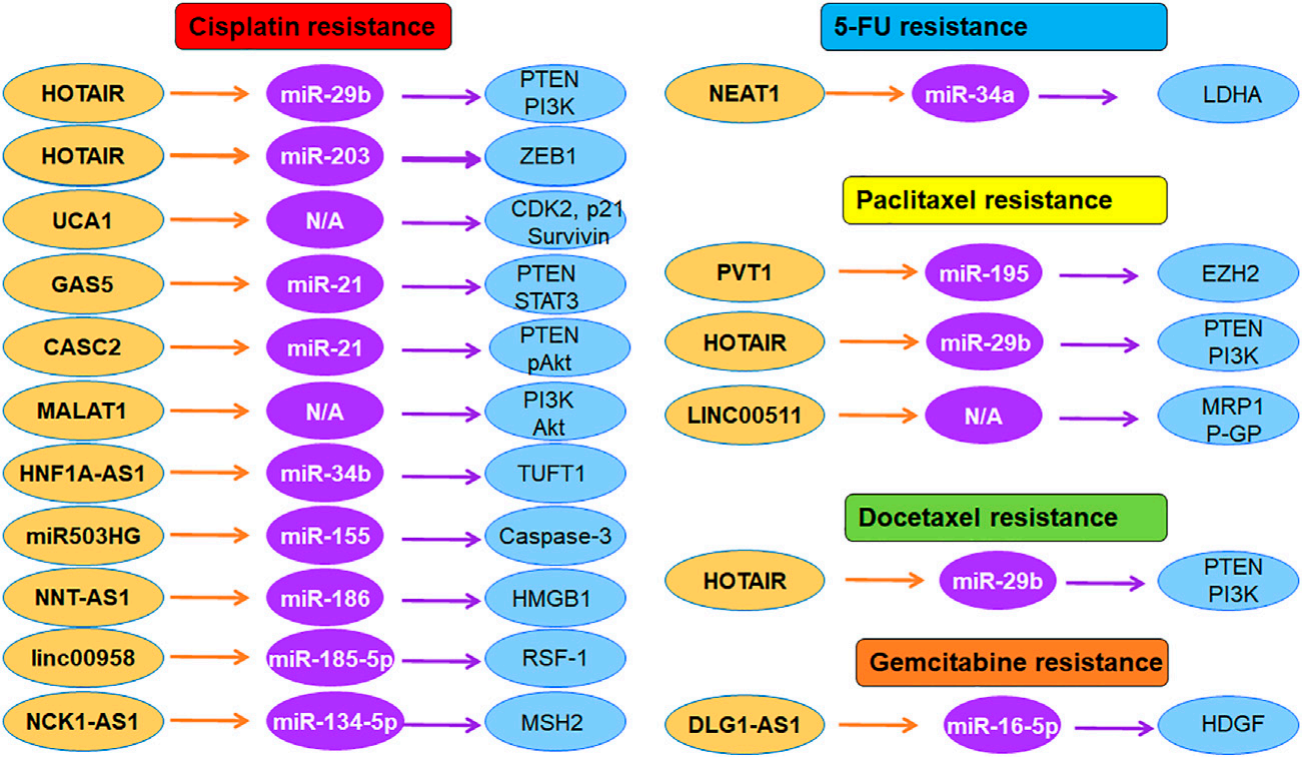
The role of lncRNAs in regulation of chemoresistance in cervical cancer. Multiple lncRNAs participate in cisplatin resistance, 5-FU resistance, paclitaxel resistance, and gemcitabine resistance in cervical cancer.

In summary, lncRNAs are critically taken part in therapeutic resistance in cervical cancer (Figure 1). Regulation of lncRNAs could overcome drug resistance to obtain good treatment benefit for cervical cancer patients. Chemotherapy is a standard approach for the treatment of cervical cancer. However, drug resistance is a huge challenge for treatment benefit. Hence, targeting lncRNAs is a good approach for reversing drug resistance. Propofol has been found to reduce tumor size and suppress cell proliferation as well as induce cell apoptosis *via* inhibition of HOTAIR and modulation of mTOR/p70S6K pathway in cervical cancer (Zhang et al., 2015). Artesunate inhibited the expression of HOTAIR and downregulated the COX-2 expression, leading to anti-metastatic ability of artesunate in cervical cancer (Zhang et al., 2016). HOTAIR interacted with COX-2 and increased COX-2 expression and catalytic activity, resulting in promotion of invasion and migration in cervical cancer (Zhang et al., 2016). Similarly, propofol repressed the cell viability, invasion, colony formation and migration in cervical cancer cells *via* targeting HOTAIR and modulating miR-129-5p/RPL14 axis (Sun et al., 2021). Further investigations are necessary to discover the compounds that regulate the expression of lncRNAs in cervical cancer.

It is critical to mention several points. Besides miRNAs and lncRNAs, circRNAs also govern drug resistance in human cervical cancer (Wen et al., 2020). Hsa_circ_0023404 promoted chemoresistance *via* VEGFA and autophagy by targeting miR-5047 (Guo et al., 2019). Moreover, circMTO1 enhanced chemoresistance *via* sponging miR-6893 in cervical cancer (Chen et al., 2019). CircMYBL2 governed paclitaxel resistance *via* targeting miR-665/EGFR axis in cervical cancer (Dong et al., 2021). Hsa_circ_0074269 increased cisplatin resistance *via* mediating miR-485-5p and TUFT1 in cervical cancer (Chen et al., 2022b). CircEPSTI1 enhanced cisplatin resistance *via* upregulation of MSH2 in cervical cancer (Wu et al., 2022). Circ_ZFR regulated paclitaxel resistance *via* sponging miR-944 and upregulating IL-10 in cervical cancer (Long et al., 2022). Depletion of circ_CEP128 increased paclitaxel sensitivity *via* targeting miR-432-5p/MCL1 in cervical cancer (Zhao et al., 2022). In addition, one lncRNA has numerous downstream targets. If one lncRNA is modulated, how can we control the expression of its numerous targets? Altogether, targeting lncRNAs by natural compounds or their inhibitors is a promising approach for overcoming drug resistance in cervical cancer.

## Abbreviations

CASC2, cancer susceptibility candidate 2; DFS, disease-free survival; DSBs, DNA double-strand breaks; EMT, epithelial to mesenchymal transition; HDGF, hepatoma-derived growth factor; HLA-G, human leucocyte antigen-G; HNF1A, human hepatocyte nuclear factor 1 homeobox A; HOTAIR, Hox transcript antisense intergenic RNA; HPV, human papillomavirus; LINP, lncRNA in non-homologous end joining pathway 1; MKL1, megakaryoblastic leukemia 1; MMP-9, matrix metalloproteinase-9; MRP, multidrug resistance protein; MSH2, Muts protein homolog 2; NEAT, nuclear-rich transcripts 1; NHEJ, non-homologous end joining; NNT-AS1, nicotinamide nucleotide transhydrogenase-antisense RNA 1; OS, overall survival; PVT1, plasmacytoma variant translocation 1; TME, tumor microenvironment; TNBC, triple-negative breast cancer; TUG1, Taurine-upregulated gene 1; VEGF, vascular endothelial growth factor; ZFAS1, zinc finger antisense 1.
